# Supplementary material for: A Critical View of the Application of the APEX Software (Aqueous Photochemistry of Environmentally-Occurring Xenobiotics) to Predict Photoreaction Kinetics in Surface Freshwaters
Source: Molecules. 2019 Dec 18;25(1):9. doi: 10.3390/molecules25010009 (PMC7017383; doi:10.3390/molecules25010009)
Supplement: Supplementary file 1 [file molecules-25-00009-s001.zip › 3.2.4_gcc-4.4.0.zip!/3.2.4_gcc-4.4.0/3.2.4_gcc-4.4.0/doc/octave/html/interpreter/Interaction-with-gnuplot.html]

Interaction with gnuplot - Untitled


Up: Graphics backends


---

##### 15.2.9.1 Interaction with `gnuplot`

— Loadable Function: val = **gnuplot\_binary** ()  
— Loadable Function: old\_val = **gnuplot\_binary** (new\_val)  
> Query or set the name of the program invoked by the plot command.
> The default value `\"gnuplot\"`. See Installation.
